# Editorial: Women in science—Rheumatology 2021

**DOI:** 10.3389/fmed.2022.1016388

**Published:** 2022-09-21

**Authors:** Silvia Piantoni, Garifallia Sakellariou

**Affiliations:** ^1^Rheumatology and Clinical Immunology Unit, Department of Clinical and Experimental Sciences, ASST Spedali Civili and University of Brescia, Brescia, Italy; ^2^Department of Internal Medicine and Therapeutics, University of Pavia, Pavia, Italy; ^3^Istituti Clinici Scientifici Maugeri IRCCS Pavia, Pavia, Italy

**Keywords:** rheumatology, research, gender equity, scientific careers, science

Even though the proportion of women in science, technology, engineering, mathematics, and medicine has arisen in the last decades, a gender imbalance among conference speakers, editors, academic positions and hiring committees still exists throughout all of these disciplines (1, 2). In fact, it was recently demonstrated that in most scientific fields men comprise more than a half of the workforce, especially at senior levels (3). Rheumatology is a medical discipline that saw a great growth in the last years. Despite the significant contribution provided by women in the research in this field, as underlined in a recent publication (4), the gender imbalance in senior academic and authorship positions is no exception (2, 5). The increase of the number of women in rheumatology is an interesting data that comes also from Arab Countries, as underlined in the first publication of our collection. Ziade et al. described the situation of the women rheumatologists' workforce in the Arab League of Associations for Rheumatology countries, underlying their increasing presence, along with their lower proportion in leadership positions and suggesting that social media platform could help them to assert themselves. In line with this universal need, in the United States, an Association of Women in Rheumatology (AWIR) was founded with the mission to promote the science and practice of rheumatology, foster the advancement and education of women in the field, and advocate access to the highest quality health care, and management of patients with rheumatic diseases^1^. The same purpose is promoted by an Italian women association in rheumatology, “Reumatologhe Donne” (ReDO)^2^.

Inspired by these principles in favor of gender equity in rheumatology, the aim of our Research Topic was to offer space to women, who are responsible of the manuscripts of this collection as first or senior authors, giving room to different scientific topics.

Women are often proactive in a multidisciplinary team, which is a fundamental need for the cure of multisystemic rheumatic conditions, as demonstrated by the paper of Schmoll et al., in which pneumologists and rheumatologists collaborated in order to cure the rheumatologic long-term complications of cystic fibrosis, or in the papers of Kramer et al., in which a multidisciplinary team studied sicca syndrome, related or not to other autoimmune symptoms, and Carmona-Fernandes et al. in which a collaboration with ^1^ vascular surgeons let rheumatologists to hypothesize the basis of the relationship between bones and vessels in the context of atherosclerotic disease and osteoporosis. Some rheumatologic conditions are very common, and a collaboration with epidemiologists is very welcome, in order to better understand relevant public health conditions, such as osteoarthritis, as reported by Costa et al. Furthermore, a collaboration with gynecologists is important for the process of counseling in view of a pregnancy, such as demonstrated by the paper of Triggianese et al.

In our collection, clinical research papers are well represented. Marinello et al. discussed the important theme of the involvement of patients in the cure of their disease in the form of shared decision making, which may be crucial for patients with rare diseases, such as Behçet's Syndrome. Mo et al. retrospectively analyzed the structural progression of the sacroiliac joint and clinical features in patients with axial spondylarthritis reducing TNF inhibitor's dose, discouraging the complete drug withdrawal. Büttner et al. proposed the use of the fluorescence optical imaging for the detection of early psoriatic arthritis.

Women are also involved in basic sciences' research. Nadali et al. explored the potential role of a soluble multiligand receptor as a new biomarker of metabolic failure developed during chronic inflammation and Kuret et al. discussed the application of the single cell RNA sequencing in the field of autoimmune diseases.

Those are examples of excellent manuscripts written under the supervision of women leaders, encompassing basic and clinical research and supporting the concept of multidisciplinary collaboration to achieve relevant results. As pointed out by a review (6), despite recent improvements in the number of women in rheumatology, further efforts must be spent to better understand and overcome causes of inequity between women and men, which still remain relevant in academia, considering that, according with a recent estimation, women are predicted to comprise the majority of the rheumatology workforce by 2025 (7, 8). A proposal of potential interventions for career advancement in academic rheumatology has just been published in order to inform an European Alliance of Associations for Rheumatology (EULAR) task force (9).

## Author contributions

SP wrote the first draft. GS edited and reviewed the draft. All authors approved the final version of the manuscript.

## Conflict of interest

The authors declare that the research was conducted in the absence of any commercial or financial relationships that could be construed as a potential conflict of interest.

## Publisher's note

All claims expressed in this article are solely those of the authors and do not necessarily represent those of their affiliated organizations, or those of the publisher, the editors and the reviewers. Any product that may be evaluated in this article, or claim that may be made by its manufacturer, is not guaranteed or endorsed by the publisher.

## Footnotes

^1^ https://rheumhighlights.com/2020/awir

^2^ https://www.reumatologhedonne.it/
